# Growth, Stoichiometry, and Palatability of *Suaeda salsa* From Different Habitats Are Demonstrated by Differentially Expressed Proteins and Their Enriched Pathways

**DOI:** 10.3389/fpls.2021.733882

**Published:** 2021-09-01

**Authors:** Ye Song, Jiayuan Liu, Jianzhong Wang, Fude Liu

**Affiliations:** ^1^College of Biological Sciences and Technology, Beijing Forestry University, Beijing, China; ^2^Jinan Fruit Research Institute, All China Federation of Supply and Marketing Co-Operatives, Jinan, China; ^3^School of Environmental Science and Safety Engineering, Tianjin University of Technology, Tianjin, China

**Keywords:** *Suaeda salsa*, differentially expressed proteins, phenylpropanoid biosynthesis, photosynthesis, medicinal and edible plant

## Abstract

*Suaeda salsa* (L.) Pall., a medicinal and edible plant, has green and red-violet ecotypes that exhibit different phenotypes, tastes, and growth characteristics. However, few studies have focused on these differences from the aspect of differentially expressed proteins under the conditions of different habitats in the field. In this study, two ecotypes of *S. salsa* from the intertidal (control) and supratidal (treatment) habitats of the Yellow River Delta were selected. A total of 30 individual leaves were mixed into six samples (three biological replicates for each) and subjected to protein extraction by using tandem mass tag-labeled quantitative proteomic technology. A total of 4771 proteins were quantitated. They included 317 differentially expressed proteins (2.0-fold change, *p* < 0.05), among which 143 were upregulated and the remaining 174 were downregulated. These differentially expressed proteins mainly participated in biological processes, such as response to stimulus, stress, and biotic stimulus; in molecular functions, such as methyltransferase activity, transferase activity, one-C group transfer, and tetrapyrrole binding; and in cell components, such as non-membrane-bound organelles, intracellular non-membrane-bound organelles, chromosomes, and photosystems. The differentially expressed proteins were mainly enriched in eight pathways, among which the ribosome, phenylpropanoid biosynthesis, and photosynthesis pathways had higher protein numbers than the other pathways. The upregulation of differentially expressed proteins related to the ribosome and photosynthesis increased the relative growth rate and reduced the *N*:*P* ratio of *S. salsa* from the supratidal habitat, thereby improving its palatability. By contrast, most of the differentially expressed proteins involved in phenylpropanoid biosynthesis were downregulated in *S. salsa* from the intertidal habitat. This result indicated that *S. salsa* from the intertidal habitat might accumulate flavonoids, lignin, and other secondary metabolites in its leaves that confer a bitter taste. However, these secondary metabolites might increase the medicinal value of *S. salsa* from the intertidal habitat. This work could provide a theoretical basis and data support for the sustainable and high-value utilization of medicinal and edible plants from coastal wetlands.

## Introduction

*Suaeda salsa* (L.) Pall. is an annual herb that belongs to the Chenopodiaceae family. It has high resistance to salinity, drought, and waterlogging. *S. salsa* is mainly distributed in Europe and Asia, including Inner Mongolia and Liaoning, Hebei, Jiangsu, and Shandong Provinces in the northeast of China. *S. salsa* is rich in amino acids, proteins, unsaturated fatty acids, dietary fiber, vitamins, minerals, and flavonoids and is a high-quality vegetable and oil crop with high medicinal value (Song and Wang, 2015; Zhao et al., 2018; Zhao Y. Q. et al., 2019). In addition, the residue of *S. salsa* seeds after oil extraction contains more than 25% crude protein and thus can be used in animal feed as a good protein source (Li and Song, 2019). Although *S. salsa* is highly edible and has high medicinal and feed value, little is known about its proteomic reflection in growth–defense tradeoffs. This situation affects its development and utilization levels in China.

Two ecotypes of *S. salsa* grow in the coastal areas of the Yellow River Delta (YRD). *S. salsa* growing in supratidal habitats is often affected by drought stress (Han et al., 2015; Liu et al., 2015). It has a plant height of 50–80 cm, luxuriant branches, fluffy shape, and green color. *S. salsa* growing in intertidal habitats is often affected by high salt, waterlogging, and low-temperature stress (Liu et al., 2015). It has a plant height of only 20–30 cm, no branches, and red-violet and fleshy leaves. In general, only the *S. salsa* with the green phenotype from supratidal habitats can be used as a vegetable, whereas the *S. salsa* with the red-violet phenotype from the intertidal habitat has low direct edible value because of its unpleasant taste. The different palatabilities of the two ecotypes of *S. salsa* may be related to the changes in N and P contents and the differential expression of the proteins in key metabolic pathways under environmental stresses. Recent studies have focused on the edible, ecological, and economic values of *S. salsa* (Liu et al., 2015; Xu et al., 2017; Zhao et al., 2018; Li and Song, 2019). However, few reports have focused on the relationships between the individual differences and growth–defense tradeoffs of *S. salsa* in different habitats.

Metabolomics and proteomics have replaced other traditional molecular marker techniques and are often used as tools to determine the expression of vegetation characteristics (Villate et al., 2021). Abiotic stress factors can directly affect the yield of plants and may cause apparent differences in plant characteristics, such as plant performance, growth, metabolism, and protein expression profiles (Wu et al., 2012; Liu et al., 2015; Wang et al., 2019, 2021). For example, in *Ocimum basilicum* L., the obvious effect of drought stress on phenylpropanoid biosynthesis increases the content of methyl tea phenol and methyl eugenol and then alters the expression profiles of leaves (Mandoulakani et al., 2017). Salt stress may regulate the synthesis of the proteins related to carbohydrates, detoxification, antioxidation, secondary metabolism, and ion transport (Rahman et al., 2015; Xiong et al., 2017; Li J. L. et al., 2020) that can enhance plant salt tolerance and subsequently improve growth performance (Zhao C. Z. et al., 2019). Other stress factors, such as pathogens and pollutants, may also lead to the overexpression of some proteins, thus changing the tolerance and external performance of plants (Feng et al., 2021). Soil nutrient conditions and distribution patterns in plant tissues are important for the expression of proteins related to plant growth and performance. For example, in cotton, P deficiency can activate the phenylpropanoid pathway, thus resulting in the transformation of protein expression profiles in cotton and enhancing resistance to verticillium wilt (Luo et al., 2021). Moreover, *N* and/or *K* deficiency may lead to the overexpression of specific enzymes and alter the transcription levels of some genes, as well as enhance the tolerance of plants for low nutrients (Li et al., 2017; Li H. et al., 2020). A previous experiment suggested that low N and P treatments increase gliadin and decrease glutenin fractions; these changes indicate that nutrient deficiency can affect the balance of protein distribution in plant tissues (Tóth et al., 2020). In addition, given that N is an important component of all enzymes and P is an important part of RNA in protein synthesis (Elser et al., 2003, 2007), the stoichiometric composition of N and P affects biological processes, such as growth, metabolism, and protein synthesis.

On the basis of the different appearances of plants and their habitats, we assumed that the regulatory proteins of ribosomal and photosynthesis pathways in the leaves of *S. salsa* from supratidal habitats are considerably upregulated. The upregulation of these proteins thus increases the relative growth rate (RGR) of *S. salsa* from supratidal habitats. However, ribosomal mRNA synthesis in fast-growing tissues requires more P than N (Elser et al., 2000; Kerkhoff et al., 2005) and can reduce the *N*:*P* ratio in leaves and subsequently increase the palatability of *S. salsa* from supratidal habitats. By contrast, the proteins involved in defense, stimulation, and stress response are significantly downregulated, indicating that phenylpropanoid biosynthesis and other secondary metabolic pathways are prevalent in intertidal habitats. We hypothesized that phenylpropanoid biosynthesis may increase the accumulation of flavonoids, lignin, and other secondary metabolites in the leaves of *S. salsa* from intertidal habitats and then directly reduce its palatability.

We selected individuals of *S. salsa* from intertidal and supratidal habitats in the YRD and used tandem mass tag (TMT) labeled quantitative proteomic technology to identify the differentially expressed proteins in leaves of *S. salsa* from different habitats and verify the above hypothesis. We aimed to establish the protein sequence database of this non-model plant species in its natural habitats, to determine the differentially expressed proteins and their regular roles in the major metabolic pathways in the leaves of *S. salsa* from different habitats, to elucidate the differences in the individual sizes and palatability of *S. salsa* on the basis of proteomics, and to determine the factors influencing the distribution of differentially expressed proteins. This research could provide theoretical and data support for understanding plant growth–defense tradeoffs in the coastal area of YRD.

## Materials and Methods

### Site Description

**FIGURE 1:**
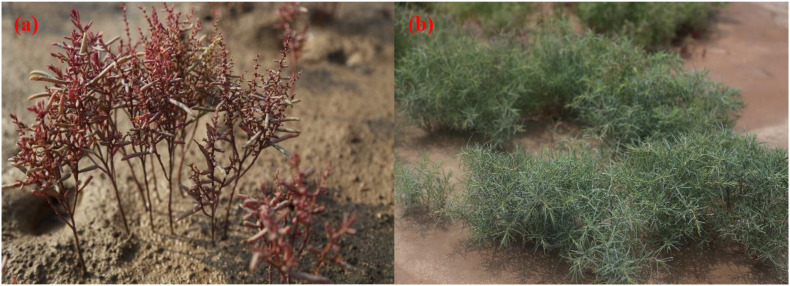
Phenotypes of *Suaeda salsa* at different habitats. **(a)** Red-violet phenotype of *S. salsa* in the intertidal habitat; **(b)** green phenotype of *S. salsa* in the supratidal habitat.

The study area is located in the YRD (E 118°57–119°20′, N 36°40′–37°50′), Dongying City, Shandong Province. This area has a warm temperate continental monsoon climate. The annual average temperature is 11.9°C, the frost-free period is 196 days, and the annual average precipitation is 640 mm. Precipitation is mostly concentrated in the summer; summer precipitation accounts for 65% of the annual precipitation. The soil in Dongying City can be divided into five types, namely, cinnamon, Shajiang black, fluvo aquic, saline, and paddy. Saline soil is distributed in the coastal area and accounts for 36% of the total soil area in Dongying City. The study area is divided into the supratidal and intertidal habitats. The individual sizes and phenotypes of *S. salsa* from different habitats are significantly different (Figure 1). The natural vegetation in the intertidal habitat is mainly *S. salsa* or the red carpet. In the supratidal habitat, the natural vegetation shrub mainly includes *Tamarix chinensis* Lour., and herbaceous plants mainly include *S. salsa*, *Limonium bicolor* (Bag.) Kuntze, *Artemisia capillaris* Thunb., *Imperata cylindrica* (L.) Beauv., *Setaria viridis* (L.) Beauv., *Phragmites communis* (Cav.) Trin. ex Steud., *Cynanchum chinense* R. Brown., and *Kochia scoparia* (L.) Schrad.

### Sampling Collection

Samples in this study were collected in August 2018. The *S. salsa* communities in the supratidal and intertidal habitats were selected as the research plots. The different phenotypes of *S. salsa* from the supratidal and intertidal habitats are shown in Figure 1. Three 1 m × 1 m quadrats were set in each plot in the intertidal and supratidal habitats. Five plants were removed from the soil in each quadrat, and the plant surface was cleaned with PBS water and dried with filter paper. Next, the leaves of *S. salsa* from each quadrat were extracted and mixed into one sample. In total, 30 individual leaves (15 red-violet phenotypes from the intertidal habitat and 15 green phenotypes from the supratidal habitat) were mixed into six samples, which were designated as Int-1, Int-2, Int-3, Sup-1, Sup-2, and Sup-3. All samples were dropped into a freezing pipe, placed quickly in a bucket of dry ice, frozen, brought back to the laboratory, and placed in a cryogenic refrigerator at −80°C for the next analysis.

Then, the soil samples were collected from the 0–10 cm soil layer under the plants by using a soil collector, placed in self-sealing bags, numbered, and brought back to the laboratory. The collected soil samples were air-dried to a constant weight in the laboratory, and impurities, such as stones, animal, and plant residues, were removed. The soil and plant samples were ground into powder by using a ball mill and then passed through a 100-mesh nylon sieve. The sieved samples were placed in self-sealing bags for subsequent chemical analysis.

### Determination of Soil Properties

Soil pH was measured via the potentiometric method with a soil:water ratio of 1:2.5 (IQ-150 Spectrum Technologies, Inc., Germany). Soil water content was measured with a soil moisture measuring instrument (HD2, IMKO, Germany). Soil salt concentration was determined by weighing at a soil:water ratio of 1:5. SOM was determined via dichromate oxidation with external heating and titration with FeH_8_N_2_O_8_S_2_. Soil total C and N concentrations were determined by using an elemental analyzer (Vario EL III, Elementar, Germany), whereas soil total P concentrations were measured through inductively coupled plasma optical emission spectrometry (Varian, Inc., United States). Total K was analyzed with a flame atom absorption spectrophotometer (TAS990SUPERAFG, Persee, China). All measurements were repeated thrice to avoid experimental errors.

### Determination of RGR

The aboveground and underground biomasses, plant height, and root length of *S. salsa* from the selected study site were measured in May, July, September, and November of 2018.

The RGR based on height (RGR_h_) and the RGR based on mass (RGR_m_) were calculated as follows:

(1) $${\mathrm{RGR}}_{m}=\frac{\mathrm{lnW2}-\mathrm{lnW1}}{\mathrm{T2}-\mathrm{T1}}$$

In Formula (1), W2 and W1 are the final and initial biomasses of *S. salsa*, respectively, in g; T2–T1 is the growth interval in months; and RGR_m_ is expressed in g g^–1^ month^–1^.

(2) $${\mathrm{RGR}}_{h}=\frac{\mathrm{lnH2}-\mathrm{lnH1}}{\mathrm{T2}-\mathrm{T1}}$$

In Formula (2), H2 and H1 are the final and initial heights of *S. salsa*, respectively, in cm; T2–T1 is the growth interval in months; and RGR_h_ is expressed in cm cm^–1^ month^–1^.

### Protein Extraction, Enzymatic Hydrolysis, and Peptide Quantification

The green phenotypes from the supratidal habitat were designated as the treatment group (Sup), and the red-violet phenotypes from the intertidal habitat were designated as the control group (Int). All samples (three biological replicates for each group) were subjected to protein extraction. Proteins were extracted with SDT (4% w/V SDS, 100 mM Tris-HCl, pH 7.6, 0.1 M DTT) and then quantified by using bicinchoninic acid. The appropriate amount of protein from each sample was digested by using trypsin via filter-assisted protein preparation, and peptides were quantified on the basis of the OD280 value.

### TMT Peptide Labeling and Grading

A total of 100 μg of peptide was obtained from each sample and labeled in accordance with the Thermo Fisher TMT labeling kit instructions. The samples were labeled as (Int-1)-126, (Int-2)-127, (Int-3)-128, (Sup-1)-129, (Sup-2)-130, and (Sup-3)-131. The labeled peptides of each group were mixed equally and graded by applying a high-pH reversed phase peptide fractionation kit. First, acetonitrile and 0.1% trifluoroacetic acid were used for column equilibrium. Then, the mixed labeled peptide samples were loaded with pure water, and low-speed centrifugation was then performed for desalination. Finally, high-pH acetonitrile solutions with increasing concentrations were used for the gradient elution of column-bound peptides. Each eluted peptide sample was dried in vacuo and then redissolved with 12 μL of 0.1% FA for liquid chromatography mass spectrometry (LC–MS) analysis. Peptide concentration was determined on the basis of the OD280 value.

### LC–MS/MS Analysis

The reconstituted peptide solution was subjected to LC–MS/MS analysis, and each grading sample was separated by Easy-nLC1200 in the HPLC liquid phase system by using a nanoscale flow rate. Buffer solution A was 0.1% formic acid in water, and buffer solution B was 0.1% formic acid in 84% acetonitrile. The chromatographic column was equilibrated with 95% A solution, and the sample was loaded into the loading column (Thermo Scientific Acclaim PepMap100, 100 μm × 2 cm, nanofiber C18) by using an automatic injector and separated in an analytical column (Thermo Scientific EASY column, 10 cm, ID 75 μm, 3 μm, C18-A2). The flow rate was 300 nL min^–1^. The samples were separated through chromatography and analyzed by using Q-Exactive MS. The detection mode was positive ion, and the scanning range for the parent ion was 300–1,800 m/z. The mass charge ratios of polypeptides and polypeptide fragments were determined by collecting 20 fragment maps (MS2 scan, higher-energy collisional dissociation) after each full scan. The resolution of primary mass spectrometry was 70,000 at 200 m/z, the automatic gain control target was 1 × 10^6^, the level 1 maximum IT was 50 ms, and the dynamic exclusion time was 60 s. The secondary mass spectrum resolution was 17,500 at 200 m/z (TMT six-plex) or 35,000 at 200 m/z (TMT 10-plex), the isolation window was 2 *m/z*, the normalized collision energy was 30 eV, and the underfill was 0.1%.

### Mass Spectrometry Data Analysis

The raw data of MS analysis were in a RAW file. Database search and identification were performed by using Mascot2.2 and Proteome Discoverer 1.4 software. The main library parameters were set as shown in Table 1.

**TABLE 1:** The main library parameters in the experiment.

Item	Value
Enzyme	Trypsin
Max missed cleavages	2
Fixed modifications	Carbamidomethyl (C), TMT 6/10 plex (N-term), TMT 6/10 plex (k)
Variable modifications	Oxidation (M), TMT 6/10 plex (Y)
Peptide mass tolerance	±20 ppm
Fragment mass tolerance	0.1 Da
Database	P20190600774_zjk_20190730.fasta
Database pattern	Decoy
Peptide FDR	≤0.01
Protein quantification	The protein ratios are calculated as the median of only unique peptides of the protein
Experimental bas	Normalizes all peptide ratios by the median protein ratio. the median protein ratio should be 1 after the normalization

### Bioinformatics Analysis

The Blast2 GO database was used to annotate the target proteins via the four following steps: blast, mapping, annotation, and annotation augmentation. The KEGG database was utilized to classify and group the identified proteins, and KEGG automatic annotation server software was applied to annotate the KEGG pathways of the target proteins. Fisher’s exact test was used to compare the distribution of the target proteins and the total proteins in each GO classification or KEGG pathway, and GO or KEGG annotation enrichment analysis was performed on the basis of the target proteins.

### Statistical Analysis

In this research, all data were statistically analyzed with SPSS 20.0. The differences in RGR_m_, RGR_h_, *N*:*P* stoichiometry, and soil properties between the intertidal and supratidal habitats were tested through one-way ANOVA. Then, *post hoc* test was performed by using least significant difference test. A heat map and volcano plot were generated with R package (R version 3.4).

## Results

### Soil Properties in Different Habitats

Soil salt and MC in the intertidal habitat were 210.42 and 97.16% higher than those in the supratidal habitat (Table 2, *p* < 0.01) and varied by 3.84–11.92‰ and 17.61–34.72%, respectively. SOM, C, N, and P are the key fertility factors that determine the growth status of organisms and were found to vary between different habitats within the ranges of 3.59–7.53, 9.98–17.00, 0.14–0.32, and 0.42–0.52 mg⋅g^−1^, respectively. In comparison with those in the supratidal habitat, SOM, soil C and soil N were significantly higher by 109.75, 70.34, and 128.57%, respectively, and TP was significantly lower by 19.23% (Table 2, *p* < 0.05) in the intertidal habitat. However, the pH and TK of the intertidal and supratidal habitats showed no significant differences.

**TABLE 2:** Soil physicochemical properties at different habitats.

Habitats	MC (%)	pH	SOM (mg.g^–1^)	Salt (‰)	TC (mg⋅g^−1^)	TN (mg.g^–1^)	TP (mg.g^–1^)	TK (mg.g^–1^)
Intertidal habitat	34.72 ± 0.61a	7.47 ± 0.32a	7.53 ± 1.34a	11.92 ± 1.59a	17.00 ± 0.79a	0.32 ± 0.03a	0.42 ± 0.02a	15.01 ± 2.25a
Supratidal habitat	17.61 ± 3.25b	7.76 ± 0.02a	3.59 ± 2.12b	3.84 ± 1.63b	9.98 ± 0.33b	0.14 ± 0.01b	0.52 ± 0.03b	14.83 ± 0.37a

*Different lower-case letters in the same column indicate significant differences (p < 0.05).*

### Growth Characteristics of *S. salsa* From Different Habitats

**FIGURE 2:**
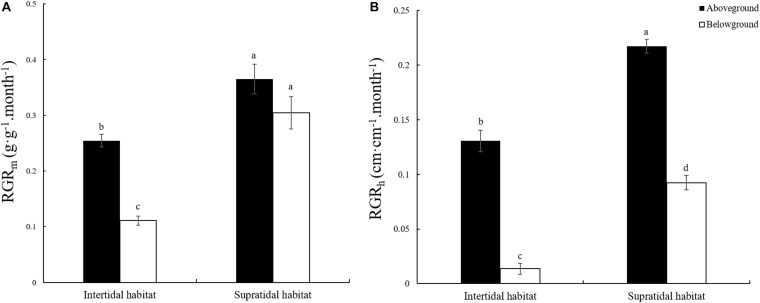
Relative growth rate (RGR) of the aboveground and belowground parts of *S. salsa* in the intertidal and the supratidal habitats. **(A)** RGR based on biomass (RGR_m_); **(B)** RGR based on height (RGR_h_). Different lower-case letters indicate significant differences (*p* < 0.05).

The RGR of plants is closely related to elemental stoichiometry. In this study, two measurement methods were adopted for RGR determination. One was based on biomass growth, and the other was based on height growth. Figure 2 shows that the RGR_m_ and RGR_h_ of the aboveground and belowground parts of *S. salsa* from the supratidal habitat were significantly higher by 43.50 and 174.33% and by 66.44 and 573.84% (Figures 2A,B, *p* < 0.05) than those of *S. salsa* from the intertidal habitat. Except for the RGR_m_ of *S. salsa* from the supratidal habitat, the RGR_m_ and all RGR_h_ of the aboveground parts were significantly higher than those of the belowground parts (Figures 2A,B, *p* < 0.05).

### *N*:*P* Stoichiometry of *S. salsa* From Different Habitats

**FIGURE 3:**
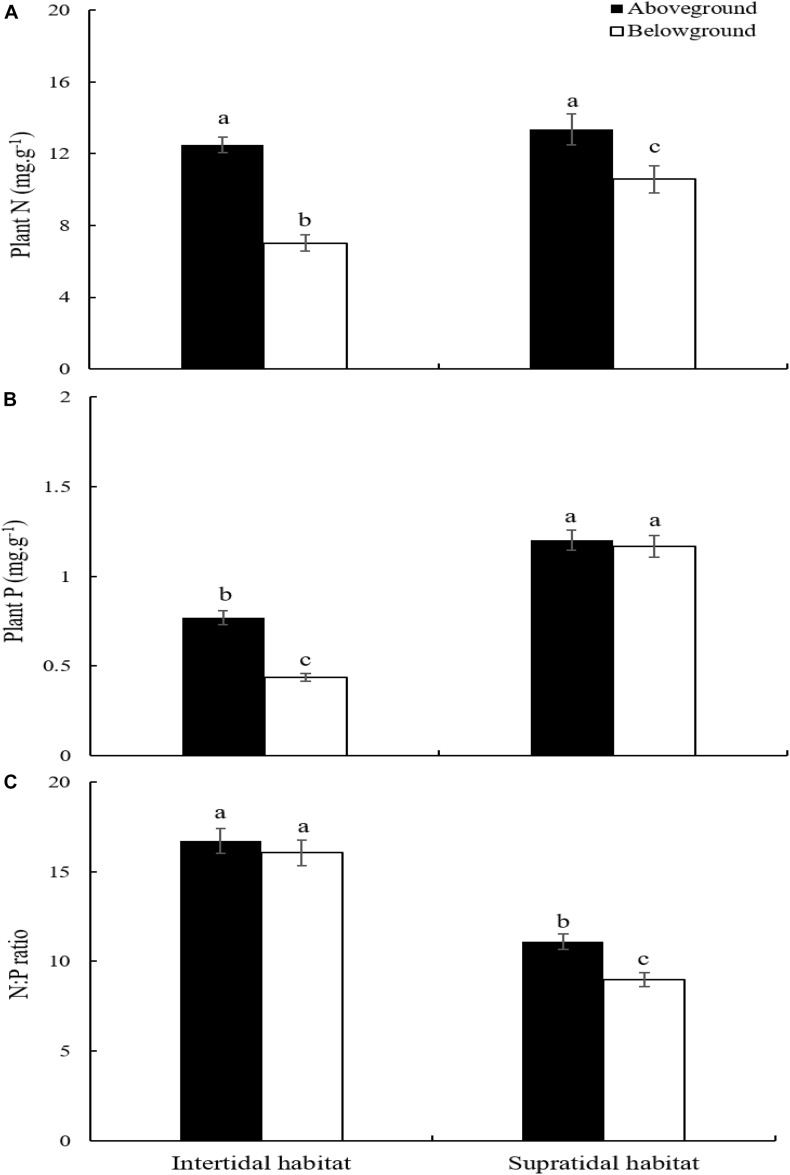
Plant *N*:*P* stoichiometry in aboveground and belowground parts of *S. salsa* at different habitat. **(A)** N concentration; **(B)** P concentration; **(C)** *N*:*P* ratio. Different lower-case letters indicate significant differences (*p* < 0.05).

*S. salsa* ecotypes from the intertidal and supratidal habitats had significantly higher N concentration in their aboveground parts than in their belowground parts (Figure 3A; *p* < 0.05). The N contents of the aboveground parts of *S. salsa* from the intertidal and supratidal habitats were not significantly different (Figure 3A; *p* > 0.05). However, the N concentration in the belowground parts of *S. salsa* from the supratidal habitat was significantly higher than that in the belowground parts of *S. salsa* from the intertidal habitat (Figure 3A; *p* < 0.05).

*S. salsa* from the intertidal habitat had significantly higher P concentration in its aboveground parts than in its belowground parts (Figure 3B; *p* < 0.05). However, no significant difference was observed between the aboveground and belowground parts of *S. salsa* from the supratidal habitat (Figure 3B; *p* > 0.05). Comparison revealed that the P concentration in the aboveground and belowground parts of *S. salsa* from the supratidal habitat was significantly higher than that in aboveground and belowground parts of *S. salsa* from the intertidal habitat (Figure 3B; *p* < 0.05).

No significant difference in *N*:*P* ratios was found between the aboveground and belowground parts of *S. salsa* from the intertidal habitat (Figure 3C; *p* > 0.05). However, *S. salsa* from the supratidal habitat had significantly higher *N*:*P* ratios in its aboveground parts (11.09) than in its belowground parts (8.97) (Figure 3C; *p* < 0.05). The *N*:*P* ratios in the aboveground and belowground parts of *S. salsa* from the intertidal habitat were 16.72 and 16.05, respectively, and were significantly higher than those in the aboveground and belowground parts of *S. salsa* from the supratidal habitat (Figure 3C; *p* < 0.05).

### Differentially Expressed Proteins of *S. salsa* From Different Habitats

**FIGURE 4:**
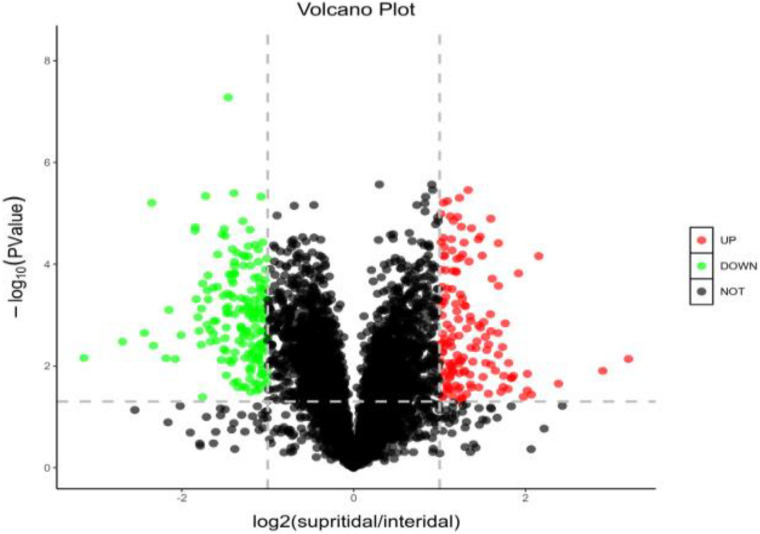
Distribution and abundance distribution of up- and downregulated proteins of *S. salsa* in different habitats; red indicates significantly upregulated differential proteins; green indicates downregulated differential proteins; black indicates no significant difference in protein.

The expressed proteins of *S. salsa* changed across different habitats (Figure 4). A total of 4,771 credible proteins were screened by mass spectrometry, and 317 differentially expressed proteins, including 143 upregulated and 164 downregulated proteins, were screened on the basis of *p* < 0.05 and change multiple ≥ 2 or ≤ 0.5. Figure 4 provides a volcano map of all the differentially expressed proteins. Black indicates proteins without significant difference, red indicates significantly upregulated proteins, and green indicates significantly downregulated proteins. The volcano map shows that a considerable number of proteins in *S. salsa* leaves had changed significantly across different habitats.

**FIGURE 5:**
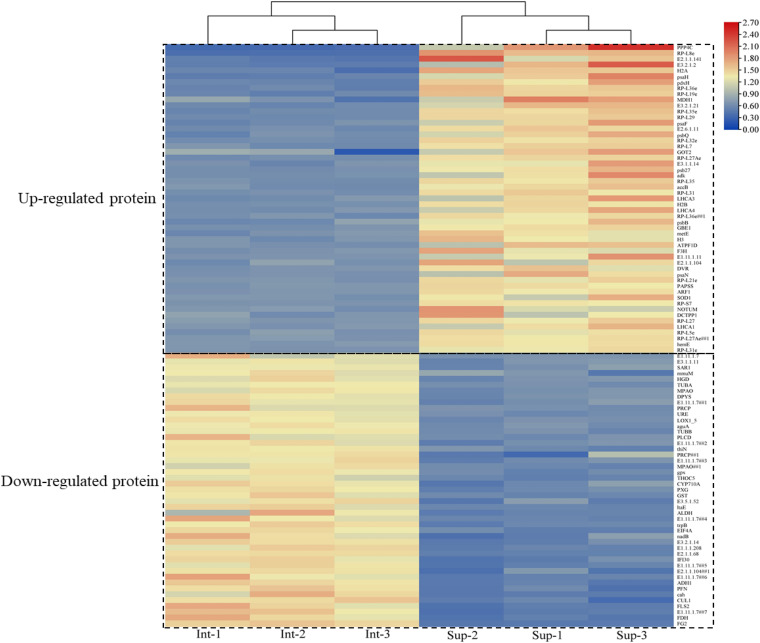
Heatmap of the identified up- and downregulated proteins at different habitats.

In total, 100 differential proteins were identified accurately through the database. They included 53 upregulated proteins and 47 downregulated proteins. The downregulated protein content in the leaves of *S. salsa* from the intertidal habitat was significantly higher than that in the leaves of *S. salsa* from the supratidal habitat. The upregulated protein content showed the opposite trend. The specific expressed proteins are shown in Figure 5.

### GO Enrichment Analysis of Differentially Expressed Proteins

**FIGURE 6:**
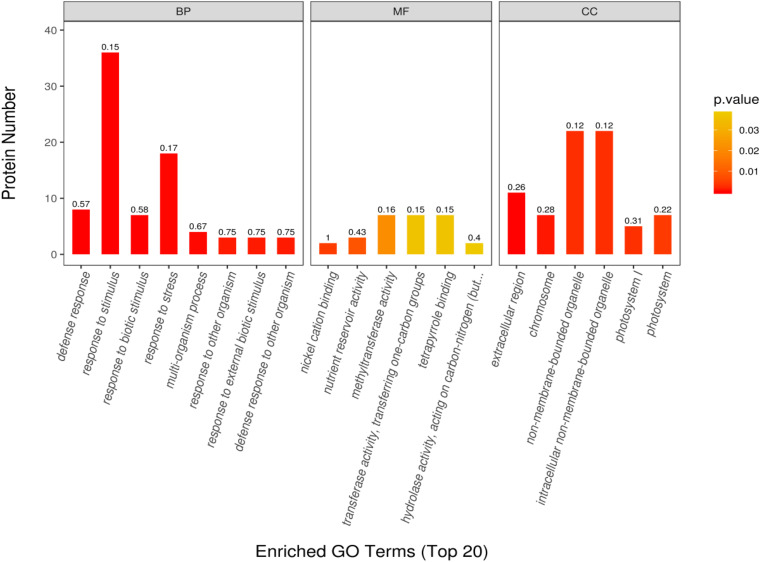
Top 20 enriched GO terms and functional classification of differential proteins.

The GO enrichment analysis was performed to determine the function of differentially expressed proteins in different habitats. Through this analysis, we found that the proteins with significant differences were mainly enriched in biological process (BP), molecular process (MF), and cell component (CC). In the BP group, the differentially expressed proteins were mainly involved in response to defense, stimulus, biological stimulus, and stress; multiorganization process; response to external biological stimulus; and defense response to other organisms. In the MF group, the differentially expressed proteins were mainly involved in Ni cation binding, nutrient reserve activity, methyltransferase activity, transferase activity transfer, one-C groups, tetrapyrrole binding, and hydrolase activity acting on C–N. In the CC group, the differentially expressed proteins were mainly involved in the extracellular region, chromosome, non-membrane-bounded organelle, intracellular non-membrane-bounded organelle, photosystem I (PSI), and photosystem II (PSII) (Figure 6). The protein numbers of BP and CC were significantly higher than those of MF.

### KEGG Pathway Enrichment Analysis

**FIGURE 7:**
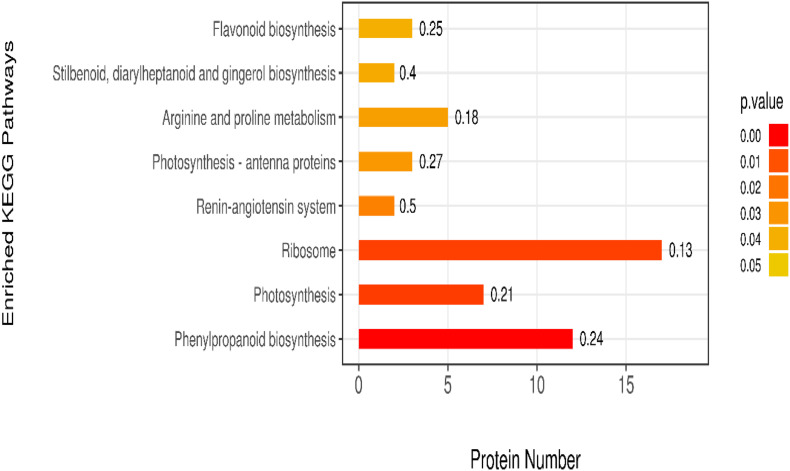
Enrichment analysis of KEGG pathway.

The KEGG is a database of known differentially expressed proteins, protein metabolism, and enriched pathways. It also contains information about metabolism, genes, human diseases, cellular processes, genetic information processing, and environmental information processing. The results showed that the differentially expressed proteins were mainly enriched in eight pathways, among which the ribosome, phenylpropanoid biosynthesis, and photosynthesis pathways had higher protein numbers than the other pathways. These three pathways had 22, 17, and 13 differentially expressed proteins. The protein numbers of flavonoid biosynthesis; stilbenoid, diarylheptanoid, and gingerol biosynthesis; arginine and proline metabolism; and photosynthesis–antenna proteins were less than 5 (Figure 7).

### Differential Protein Analysis Based on KEGG Pathway Enrichment

We uploaded the differential proteins to the KEGG website to annotate sequences and metabolic pathways to further understand the metabolic pathway information of *S. salsa* in different habitats. A total of 51 differential proteins were annotated within the range of *p* < 0.05 into eight KEGG pathways. The first three KEGG pathways with the most significant differences were the ribosome, phenylpropanoid biosynthesis, and photosynthesis pathways.

**FIGURE 8:**
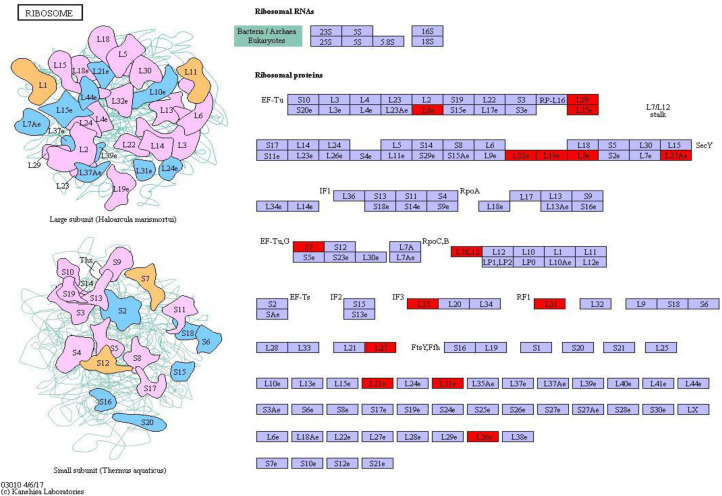
Ribosome pathway and differential protein expression. The red box indicates upregulation of differentially expressed proteins.

Ribosomal protein plays an important role in regulating protein synthesis and maintaining the stability of ribosomal metabolites in microorganisms. On the basis of the metabolic pathway information obtained under environmental stresses, the pathway with the largest number of differential proteins was ribosome (map_ ID: ko03010), and 22 ribosomal proteins were significantly upregulated (Figure 8). Therefore, most of the proteins were upregulated in *S. salsa* from the supratidal habitat, and the ribosomal proteins in the leaves of *S. salsa* from the supratidal habitat were significantly higher than those in the leaves of *S. salsa* from the intertidal habitat. Hence, the protein synthesis capability in the leaves of *S. salsa* from the supratidal habitat was high, whereas the protein expression capability of the plants from the intertidal habitat decreased significantly under environmental stresses. Among the upregulated proteins, Rp-L8e, RP-L36e, and RP-l19e had the highest fold-changes of 4.45, 2.82, and 2.77, respectively.

**FIGURE 9:**
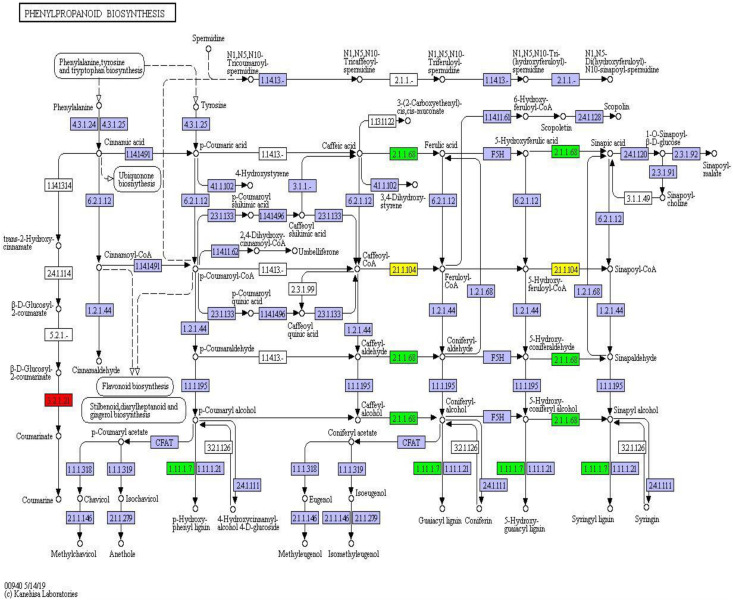
Phenylpropanoid synthesis pathway and differential protein expression. The red box represents the upregulated protein; the green box represents the down-regulated protein; the yellow box contains both up- and downregulated proteins.

Twelve differentially expressed proteins belonged to the phenylpropanoid component and were fixed in the phenylpropanoid synthesis pathway (map_ ID: ko00940). Peroxidase (1.11.1.7) and caffeic acid 3-methyl-methyltransferase (2.1.1.68) were downregulated proteins. Caffeoyl coenzyme, a methyl methyltransferase, was up- and downregulated, and β-glucosidase was upregulated (Figure 9). These results indicated that phenylpropanoid synthesis had different expression profiles in different habitats. Peroxidase, coenzyme, and methyltransferase were overexpressed under environmental stress conditions. However, the expression of β-glucosidase was weakened.

**FIGURE 10:**
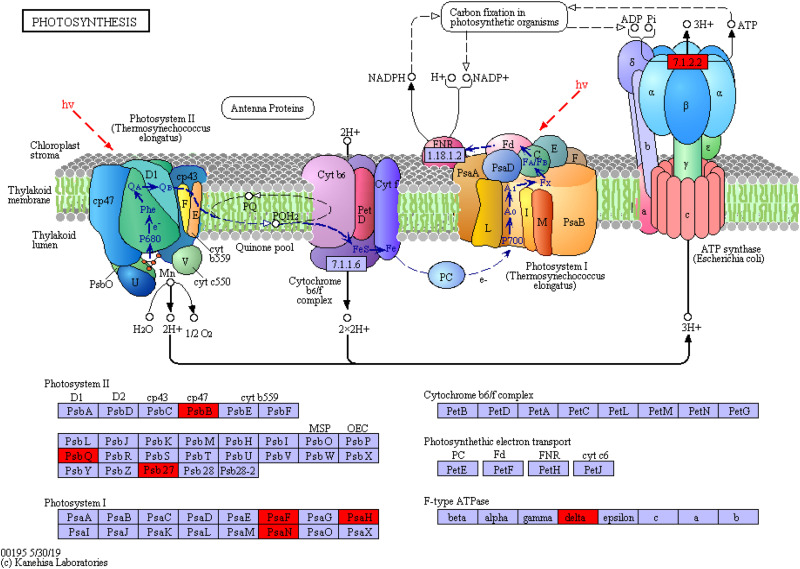
Photosynthetic protein synthesis pathway and differential protein expression. The red box represents the upregulated protein.

In addition, the proteins involved in photosynthesis were enriched in the photosynthesis pathway (map_ ID: ko00195, Figure 10). These proteins included PSI and PSII, cytochrome b6/f complex, photosynthetic electron transport, and F-type ATPase. The significant upregulation of chlorophyll apolipoprotein (PsbB), acetylguanine aminotransferase (PsbQ), and Psb27 protein (Psb27) in PSII; the PSI subunit proteins PsaF and PsaH and the PsaN protein (PsaN) in PSI; and the delta protein in F-type ATPase indicated that the protein and functional expression profiles significantly differed between the supratidal and intertidal habitats. The overexpression of proteins reflected the high growth rate of *S. salsa* from the supratidal habitat in terms of height and biomass. However, the expression of proteins involved in photosynthesis decreased as a result of high salinity, low temperature, and tidal effect in the intertidal habitat. This reduction resulted in the decrement in the photosynthetic capacity and hence the RGR of *S. salsa* from the intertidal habitat.

### Effects of Soil Properties on the Differentially Expressed Proteins

**FIGURE 11:**
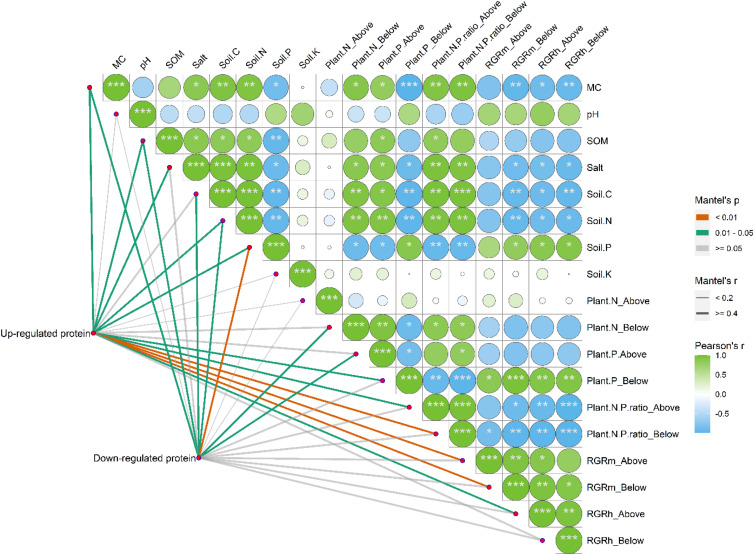
Pairwise comparisons between environmental parameters and the up-regulated and down-regulated proteins of *S. salsa* under different habitats. The up-regulated and down-regulated proteins were correlated to each environmental parameter by partial Mantel tests. Line width indicates the Mantel’s *r* statistic for the corresponding correlation coefficient, and line color represents the statistical significance. The color gradient and square represent Spearman’s correlation coefficients.

Correlation analysis suggested that the environmental parameters of MC, SOM, salinity, soil C, and soil N were positively and significantly correlated with each other (Figure 11, *p* < 0.05). However, all these parameters were found to be negatively and significantly correlated with soil P (*p* < 0.05). Notably, soil K and pH had no significant correlations with other environmental parameters (*p* > 0.05). When the upregulated and downregulated proteins were included in the study on the correlations between environmental parameters and the differentially expressed proteins of *S. salsa* from different habitats, we found that MC; SOM; salt content; and soil C, N, and P had significant effects on the up- and/or downregulated proteins of *S. salsa* ecotypes from different habitats (*p* < 0.05). Among all environmental parameters, soil P was the most important factor that affected the downregulated proteins in *S. salsa* from the intertidal habitat.

The heatmap also showed that the RGR_m_ of the belowground organs and the RGR_h_ of the aboveground and belowground organs of the *S. salsa* plants were significantly and positively correlated with soil P but were negatively correlated with soil C and soil N (Figure 11, *p* < 0.05). The RGR_m_ and RGR_h_ of the aboveground and belowground organs of plants were positively affected by root P and negatively affected by root and shoot N/P ratios (*p* < 0.05). Notably, plant growth traits were mainly affected by the root P concentrations and *N*:*P* ratios of whole plants, which were strongly correlated with upregulated proteins in leaves. However, downregulated proteins were found to be correlated with N concentrations in the roots and P concentrations in the shoot, which had weak effects on plant growth traits.

## Discussion

Protein, one of the basic substances of plant cells, plays an important role in promoting plant growth and coping with environmental changes. The overexpression of proteins in a certain environment enhances the adaptability of plants to the external environment (Jami et al., 2008; Divya et al., 2010; Zhang et al., 2021). In plants, environmental stress can promote secondary metabolite biosynthesis, which consequently reduces palatability and the probability of being eaten by increasing the accumulation of astringent, bitter, and sour substances, such as tannins, alkaloids, and terpenoids (Tátrai et al., 2016; Holopainen et al., 2018). In the present study, we analyzed the GO functional annotation and KEGG pathways of differentially expressed proteins by using TMT proteomics technology. On the basis of the differentially expressed proteins in the leaves, we found that *S. salsa* from different habitats adapted to environmental changes mainly by regulating ribosome biosynthesis, photosynthesis, and phenylpropanoid metabolism. Therefore, *S. salsa* showed different growth patterns and properties in different habitats.

### Upregulated Proteins in *S. salsa* From the Supratidal Habitat

The ribosome is the most important apparatus of protein synthesis in cells, especially rapidly proliferating cells (Zhang X. J. et al., 2020; Zhang X. X. et al., 2020). We screened 143 upregulated differential proteins, among which 53 were accurately identified. Among these proteins, the large subunit ribosomal protein, subunit protein of PSI, PsaN, PsbB, PsbQ, Psb27 in PSII, and delta protein in F-type ATPase were the major differentially expressed proteins involved in the ribosome pathway. These proteins were significantly higher in the leaves of *S. salsa* from the supratidal habitat than in in the leaves of *S. salsa* from the intertidal habitat. Their important roles in regulating the ribosome biosynthesis pathway and photosynthesis accounted for the higher RGR_h_ and RGR_m_ of *S. salsa* from the supratidal habitat than those of *S. salsa* from the intertidal habitat. The growth rate hypothesis states that for plants in natural ecosystems, high RGRs are associated with low *N*:*P* ratios (Kerkhoff et al., 2005). Considering that the synthesis of ribosomal mRNA in fast-growing tissues requires more P than N, the *N*:*P* ratios in leaves decreases, and the palatability of plants increases. The *N*:*P* ratios of the aboveground and belowground parts of *S. salsa* from the supratidal habitat were 11.09 and 8.97, respectively, and were significantly lower than those of the aboveground and belowground parts of *S. salsa* from the intertidal habitat (16.72 and 16.05 for the aboveground and belowground parts, respectively). Therefore, the high RGR and low *N:P* ratio of *S. salsa* from the supratidal habitat suggested that plants from this habitat are palatable.

The metabolic pathways of the ribosome and photosynthesis may also be affected by environmental factors. Although PSII may inhibit the function and expression of photosynthetic bacteria, the upregulation of the PSI reactive protein can compensate for the effects of PSII and enhance the photosynthetic expression capability of plants (Liu et al., 2021). This finding supported the significantly higher RGR_m_ of *S. salsa* from the supratidal habitat than that of *S. salsa* from the intertidal habitat. The production of amino acids and other organic compounds by plants during respiration leads to the production of ATP in the mitochondria and can be accompanied by the production of energy during protein synthesis (Tcherkez et al., 2020). Photosynthesis and ribosome synthesis are two important metabolic processes in plants; their interaction requires the sharing of energy intermediates and is crucial for plant growth and energy metabolism (Analin et al., 2020). In plants, salt stress affects the main processes of growth, such as germination, photosynthesis, nutrient transport, or production metabolism, and ultimately reduces the rate of vegetative growth (Dinakar et al., 2010a, b). In the present study, soil salt content in the supratidal habitat was obviously lower than that in the intertidal habitat and dramatically affected the distribution of upregulated proteins. Therefore, low salt was beneficial to the growth and metabolism of *S. salsa* from the supratidal habitat. The differential regulatory proteins involved in the ribosome and photosynthesis are mainly subunit proteins, and P is an important component of RNA in protein synthesis (Elser et al., 2003, 2007). The results showed that the P content in the supratidal habitat was higher than that in the intertidal habitat. Given that the distribution of upregulated proteins was significantly affected by soil P (*p* < 0.05), the high P condition of the supratidal habitat was conducive to improving the efficiency of ribosomal protein synthesis and increasing the growth rate and palatability of *S. salsa*.

### Downregulated Proteins in *S. salsa* From the Intertidal Habitat

The change in salinity has different effects on signal transduction, genetic information processing, protein expression, and various metabolic pathways at different growth stages of plants, including germination, growth, and dead leaf stage (Patel et al., 2016; Chen et al., 2019). Recent studies have shown that phenylpropanoid metabolism plays an important role in plant response to environmental stress (Bhuiyan et al., 2009; Bonawitz et al., 2014; Zhang et al., 2021). In the present study, the differential proteins in the leaves of *S. salsa* from the intertidal and supratidal habitats indicated that the increase in salinity enhanced the expression of peroxidase and caffeic acid 3-methyl-methyltransferase in leaves but weakened that of β-glucosidase. The overexpression of peroxidase and methyltransferase in leaves may be an adaptive strategy of *S. salsa* under salt and waterlogging stresses in intertidal habitats. The overexpression of methyltransferase may increase under drought or salt stresses, thereby enhancing the capability of plants to adapt to environmental changes (Mandoulakani et al., 2017). This finding is consistent with our result showing that the differential regulatory proteins related to the phenylpropanoid pathway were overexpressed in the leaves of *S. salsa* from the intertidal habitat in response to high salt and waterlogging stresses. In addition, the major differentially expressed proteins in the phenylpropanoid pathway were different from those in the ribosome and photosynthesis pathways, and most of the regulatory proteins in the phenylpropanoid pathway were enzymes. N is the key component of all enzymes (Elser et al., 2003, 2007). Thus, the high concentrations of soil N and SOM in the intertidal habitat may significantly promote phenylpropanoid biosynthesis in *S. salsa* from this habitat.

Plants have numerous metabolic pathways, and the enrichment of proteins in different metabolic pathways allows plants to adapt to drastic changes in the external environment. Some metabolic pathways in plants may change under salt stress (Raghavendra et al., 2010; De Zelicourt et al., 2016). For example, the phenylpropanoid biosynthesis pathway is very important in the plant growth process (Tcherkez et al., 2020). Metabolites related to the phenylpropanoid pathway, including coumarin, phenylpropanoids, lignans, and lignin, and other secondary metabolites accumulate during plant growth (Fraser and Chapple, 2011). Lignin is a complex phenolic polymer that is formed by three kinds of monolignol monomers (*p*-coumaryl alcohol, conifery alcohol, and sinapyl alcohol). It is one of the components of the plant cell wall and has functions in cell connection. Lignin fills in the cellulose framework to enhance the mechanical strength of the plant body; this effect is conducive to water transport and resistance to external environment stresses (Degenhardt et al., 2000; de Lima et al., 2014). The higher salt content of the soil in the intertidal habitat than that in the supratidal habitat may increase the mechanical strength of cells of *S. salsa* from the intertidal habitat. Cotton is well known to increase its content of flavonoids and lignin via the phenylpropanoid pathway to increase its defense capability under salt stress (Luo et al., 2021). The downregulated proteins in *S. salsa* from the intertidal habitat were more affected by soil MC and P concentrations than by salt content. The increase in the mechanical strength of plant cells is conducive to resistance to flooding, storms, and herbivory. In *S. salsa*, the accumulation of flavonoids, lignin, and other secondary metabolites would increase the astringency and bitterness of edible parts and thus reduce palatability. Notably, considering that secondary metabolites, such as flavonoids, phenolic compounds, and terpenoids, can be used as medicinal components, this effect will enhance the medicinal values of *S. salsa* from the intertidal habitat.

## Conclusion

The differentially expressed proteins of the ribosome and photosynthesis pathways were significantly upregulated in the leaves of *S. salsa* from the supratidal habitat. The upregulation of these proteins increased the RGR and reduced the *N*:*P* ratios of *S. salsa*. Subsequently, the palatability of *S. salsa* from the supratidal habitat was enhanced. The differential proteins involved in defense, stimulation, and stress response in *S. salsa* from the intertidal habitat were significantly higher than those in *S. salsa* from the supratidal habitat. These regulatory proteins promoted the biosynthesis of phenylpropanoids and other secondary metabolites. The accumulation of flavonoids, lignin, and other secondary metabolites in leaves directly reduced the palatability of *S. salsa* but significantly increased the medicinal value of this plant. This finding can provide a theoretical basis and data support for the sustainable and high-value utilization of medicinal and edible plants from the coastal wetland of the YRD.

## Data Availability Statement

The datasets generated for this study can be found in online repositories. The names of the repository/repositories and accession number(s) can be found below: http://www.proteomexchange.org/, PXD027682.

## Author Contributions

YS and FL designed the experiments. YS, JL, and FL performed the experiments, analyzed the data, and wrote the manuscript. JW and FL revised the manuscript. All authors contributed to the article and approved the submitted version.

## Conflict of Interest

The authors declare that the research was conducted in the absence of any commercial or financial relationships that could be construed as a potential conflict of interest.

## Publisher’s Note

All claims expressed in this article are solely those of the authors and do not necessarily represent those of their affiliated organizations, or those of the publisher, the editors and the reviewers. Any product that may be evaluated in this article, or claim that may be made by its manufacturer, is not guaranteed or endorsed by the publisher.
